# Comprehensive Evaluation of Fontan-Associated Liver Disease: The Role of Magnetic Resonance Imaging

**DOI:** 10.3390/diagnostics16213512

**Published:** 2026-10-27

**Authors:** Diego B. Ortega-Zhindón, Ruth V. Nuñez-Bupunari, Juan Calderón-Colmenero, Regina de la Mora-Cervantes, Daniel Campuzano-González, Nonanzit Pérez-Hernández, José Manuel Rodríguez-Pérez, Gabriela Meléndez-Ramirez, José A. García-Montes, Jorge L. Cervantes-Salazar

**Affiliations:** 1 Department of Pediatric Cardiac Surgery and Congenital Heart Disease, Instituto Nacional de Cardiología Ignacio Chávez, Mexico City 14080, Mexico; diegob.ortegaz@gmail.com (D.B.O.-Z.); ruth.nunez@uabc.edu.mx (R.V.N.-B.); 2 Department of Pediatric Cardiology, Instituto Nacional de Cardiología Ignacio Chávez, Mexico City 14080, Mexico; juanecalderon@yahoo.com.mx; 3 Department of Cardiac Tomography, Instituto Nacional de Cardiología Ignacio Chávez, Mexico City 14080, Mexico; reginadelamora@hotmail.com; 4 Department of Magnetic Resonance Imaging, Instituto Nacional de Cardiología Ignacio Chávez, Mexico City 14080, Mexico; danicampuzano1@hotmail.com (D.C.-G.); gabrielamelram@yahoo.com.mx (G.M.-R.); 5 Department of Molecular Biology, Instituto Nacional de Cardiología Ignacio Chávez, Mexico City 14080, Mexico; unicanona@yahoo.com.mx (N.P.-H.); josemanuel_rodriguezperez@yahoo.com.mx (J.M.R.-P.); 6 Department of Interventional Cardiology in Congenital Heart Disease, Instituto Nacional de Cardiología Ignacio Chávez, Mexico City 14080, Mexico; pepegamon@yahoo.com.mx

**Keywords:** Fontan procedure, Fontan-associated liver disease, cardiovascular magnetic resonance imaging, liver fibrosis, congenital heart disease

## Abstract

**Background:** Fontan-associated liver disease (FALD) develops as a consequence of chronic systemic venous congestion, which alters hepatic hemodynamics and promotes sinusoidal congestion, inflammation, and fibrosis. Our objective was to evaluate liver changes using magnetic resonance imaging (MRI) in patients with a Fontan circuit. **Methods:** We conducted a retrospective study including patients who underwent the Fontan procedure between January 2005 and December 2019 and were evaluated using T1, T2 and apparent diffusion coefficient mapping. Patients were classified into two groups: none-to-mild FALD (≤5 points) and moderate-to-severe FALD (≥6 points). FALD severity was characterized, and logistic regression analysis was performed to evaluate the association between the variables of interest and the outcome. The discriminative performance of quantitative MRI parameters was also assessed using ROC curves. **Results:** Thirty-nine patients were included, with a median age at surgery of 9.3 years (IQR 5.9–10.8); 61.5% (n = 24) were female. The most frequent diagnosis was tricuspid atresia (51.3%, n = 20), and the Fontan procedures were mainly performed using an extracardiac conduit (97.4%, n = 38). No factors were significantly associated with moderate-to-severe FALD. T1 mapping of segment I demonstrated the highest discriminative performance (AUC = 0.771; 95% CI: 0.500–1.000), with a cutoff value of >734 ms, yielding a sensitivity of 83.3% (95% CI: 43.65–99.15) and a specificity of 75% (95% CI: 57.89–86.75). **Conclusions:** After 10 years of follow-up, 15% had moderate-to-severe FALD. T1 mapping of segment I demonstrated the highest discriminative performance, with a sensitivity greater than 80% for identifying moderate-to-severe FALD.

## 1. Introduction

The Fontan procedure represents the final stage of palliative treatment for patients with congenital heart disease affected by a complex cardiac anomaly known as univentricular heart and has dramatically improved survival in this population [1,2,3,4]. Since its original description, the procedure has undergone several modifications; however, its primary objective remains to derive the systemic venous return to the pulmonary arteries without the assistance of a subpulmonary ventricle, thereby establishing passive pulmonary blood flow and serial circulation [5,6,7,8,9,10]. Although this strategy has substantially improved life expectancy and quality of life, the persistence of chronically elevated systemic venous pressure and chronic alterations in end-organ perfusion predispose these patients to the development of long-term multisystem complications [11,12,13,14,15].

Fontan-associated liver disease (FALD) is one of the most important extracardiac complications and constitutes a hallmark of late Fontan failure [13,14,15,16]. This progressive complication results from chronic systemic venous congestion, reduced portal venous flow, and altered hepatic perfusion inherent to the Fontan circulation, all of which promote hepatic inflammation, fibrosis, and, ultimately, cirrhosis and hepatocellular carcinoma in advanced stages [13,14,16,17,18,19]. Hepatic injury has been shown to develop subclinically during the early stages after Fontan completion and progress with increasing postoperative follow-up.

Consequently, systematic surveillance using noninvasive modalities has become essential. Although liver biopsy remains the reference standard for the assessment of hepatic fibrosis, its invasive nature limits its routine clinical use. Therefore, imaging modalities such as ultrasonography with liver elastography and non-contrast magnetic resonance imaging (MRI) have emerged as promising tools for the detection and longitudinal monitoring of hepatic injury, allowing safe, reproducible, and serial assessment of these patients [13,14,16,17,18,19].

Therefore, the aim of this study was to evaluate liver changes using MRI in patients who had undergone the Fontan procedure.

## 2. Materials and Methods

A retrospective, observational, analytical study was conducted in patients who underwent the Fontan procedure at the Instituto Nacional de Cardiología Ignacio Chávez between 1 January 2005 and 31 December 2019, before reaching 18 years of age. Eligible patients were required to be alive at the time of evaluation, have no history of Fontan takedown, and have available laboratory results, abdominal ultrasonography, and at least one follow-up assessment incorporating both cardiac and liver MRI. Patients who underwent the Fontan procedure at another institution, could not be contacted or did not attend follow-up visits, or had unavailable or suboptimal abdominal ultrasonography and cardiac and liver MRI studies were excluded (Figure 1). Clinical data were retrieved from medical records. The study was approved by the institutional review board (CI-116-2025), which waived the requirement for informed consent.

Collected demographic and clinical variables included primary diagnosis, predominant ventricular morphology, NYHA functional class [20], previous surgeries, catheterization history, and preoperative anatomical and hemodynamic findings from echocardiography and cardiac catheterization. Intraoperative variables included CPB and aortic cross-clamping use and duration, cardioplegia type, and conduit and fenestration size. Postoperative and follow-up variables comprised pediatric intensive care unit (PICU) length of stay, duration of mechanical ventilation, duration and volume of thoracic and peritoneal drainage, hemodynamic parameters assessed by echocardiography, cardiac catheterization, cardiac MRI, and early and late postoperative complications.

### 2.1. Liver Magnetic Resonance Imaging Protocol

The liver MRI studies were performed using a 1.5-T scanner (Magnetom Avanto; Siemens Healthcare, Erlangen, Germany) at the Department of Magnetic Resonance Imaging of the Instituto Nacional de Cardiología Ignacio Chávez. Following a previously standardized imaging protocol [17,19], complete coverage of the hepatic parenchyma was achieved.

Morphological assessment was performed using Half-Fourier Acquisition Single-shot Turbo Spin-Echo (HASTE) sequences and three-dimensional Volumetric Interpolated Breath-hold Examination (VIBE) gradient-echo sequences acquired during breath-holding. Quantitative relaxation times were measured using a Shortened Modified Look-Locker Inversion Recovery (ShMOLLI) sequence for T1 mapping and a True Fast Imaging with Steady-State Precession (TrueFISP/TRUFI) based sequence for T2 mapping. Both sequences were acquired during breath-holding with electrocardiographic (ECG) gating. Diffusion-weighted imaging (DWI) was performed using b-values of 50 and 800 s/mm^2^, from which apparent diffusion coefficient (ADC) maps were generated.

Quantitative T1 and T2 maps were processed using commercially available software (Syngo InSpace Dynamic, software platform syngo.via; Siemens Healthcare, Erlangen, Germany), whereas ADC maps were generated and analyzed on a dedicated workstation (IPAX EE; Agfa HealthCare, Bonn, Germany). To obtain regional measurements, T1 and T2 relaxometry maps, as well as ADC maps, were manually segmented according to the Couinaud liver segmentation system [21]. Acquisition time for the quantitative sequences ranged from 4 to 8 min, depending on the patient’s body size. No contrast medium was used in any study. (Figure 2).

### 2.2. Assessment and Classification of Fontan-Associated Liver Disease

For the assessment of FALD, a previously validated liver disease score (LDS) [17,19] was calculated using laboratory parameters and findings from abdominal ultrasonography. Laboratory values were evaluated according to age- and sex-specific reference ranges [22,23] and included platelet count, gamma-glutamyl transferase (GGT), aspartate aminotransferase (AST), serum albumin, and prothrombin time (PT). One point was assigned for the presence of each of the following abnormalities: thrombocytopenia, hypoalbuminemia, elevated GGT, elevated AST, or prolonged PT. PT was not considered abnormal in patients receiving oral anticoagulant therapy.

Abdominal ultrasonography findings were scored as follows: parenchymal fibrotic changes of the liver (absent, 0 points; mild, 1 point; moderate, 2 points; severe, 3 points), blunt liver edge (1 point), surface nodularity (1 point), splenomegaly (1 point), and ascites (1 point). The maximum LDS score was 12 points. Liver disease severity was stratified into four categories: none (0–2 points), mild (3–5 points), moderate (6–8 points), and severe (9–12 points). Finally, for analytical purposes, the cohort was dichotomized into two severity groups (Table 1): none-to-mild FALD (≤5 points) and moderate-to-severe FALD (≥6 points).

### 2.3. Statistical Analysis

Descriptive statistics were used to summarize study variables. Categorical variables were reported as frequencies and percentages and continuous variables as mean ± standard deviation (SD) or median and interquartile range (IQR), according to their distribution. Normality was assessed using histograms, Q–Q plots, the Shapiro–Wilk test, and the skewness–kurtosis test.

Interobserver agreement was assessed using Cohen’s kappa (κ) for categorical variables (0.61–0.80, substantial; ≥0.81, excellent) and the intraclass correlation coefficient (ICC) for continuous variables (0.75–0.90, good to excellent; ≥0.91, almost perfect).

Between-group comparisons (none-to-mild vs. moderate-to-severe FALD) were performed using the chi-square (χ^2^) test or Fisher’s exact test for categorical variables and the independent-samples Student’s *t*-test or Mann–Whitney U test for continuous variables, as appropriate. Associations with FALD severity were examined using univariable and multivariable logistic regression, with effects reported as odds ratios (ORs) and 95% confidence intervals (95% CIs).

The discriminative performance of liver MRI parameters (T1 and T2 relaxation times and ADC) was assessed using receiver operating characteristic (ROC) curves and the area under the curve (AUC). Optimal cutoffs were determined using the Youden index to identify thresholds maximizing the combined sensitivity and specificity. All tests were two-sided, with *p* < 0.05 considered statistically significant. Analyses were performed using Stata 17 (StataCorp LLC, College Station, TX, USA) and R 3.1.1 (R Foundation for Statistical Computing, Vienna, Austria).

The anatomical illustration of the liver was manually designed by the author R.V.N.-B. using Microsoft PowerPoint (Microsoft Corporation, Redmond, WA, USA; version 2016). Subsequently, for the preparation of the anatomical segments shown in Figure 3, the generative artificial intelligence tool Claude (Anthropic, Claude Sonnet 5 model) was used exclusively to generate the code employed for color assignment to the hepatic segments. Color coding was performed by linear interpolation of the numerical T1, T2, and ADC values corresponding to each segment within the minimum–maximum ranges observed in the dataset, using a progressive color scale from green to yellow, orange, and red.

## 3. Results

### 3.1. Clinical-Demographic Characteristics

Between January 2005 and December 2019, 173 Fontan procedures were performed at our institution, representing 3.4% of the 5100 cardiac surgeries performed during this period. Of these, 39 patients met the study inclusion criteria (Figure 1); 38.5% (n = 15) were male and 61.5% (n = 24) were female. At surgery, the median age was 9.3 years (IQR 5.9–10.8), weight 25 kg (IQR 18.5–37.5), and body surface area 0.9 m^2^ (IQR 0.8–1.2), with no significant differences according to FALD severity (none-to-mild vs. moderate-to-severe). Preoperatively, 76.9% (n = 30) were NYHA functional class II. The most common diagnoses were tricuspid atresia (51.3%, n = 20) and double outlet right ventricle (12.8%, n = 5), followed by pulmonary atresia with intact ventricular septum, transposition of the great arteries, and other complex congenital heart diseases (Table 1 and Table 2). Regarding previous surgical history, 22 patients (56.4%) had undergone one prior procedure and 12 (30.8%) two; the most frequent procedures were bidirectional cavopulmonary connection (58.9%, n = 23) and systemic-to-pulmonary shunt (48.7%, n = 19) (Table 1 and Table 2). All patients underwent preoperative cardiac catheterization as part of the surgical protocol.

Among preoperative anatomical and hemodynamic characteristics, the predominant ventricular morphology was left (74.4%, n = 29), followed by right (15.4%, n = 6) and indeterminate morphology (10.3%, n = 4), with no significant differences between groups (*p* = 0.13) (Table 2). Median arterial oxygen saturation was 90% (IQR 85–94), and median systemic ventricular ejection fraction was 61% (IQR 60–64), without significant variations according to FALD severity (Table 2). Regarding hemodynamic profile, the median systemic ventricular end-diastolic pressure (SVEDP) was 8 mmHg (IQR 7–10), and the median of mean pulmonary artery pressure (mPAP) was 12 mmHg (IQR 10–13). None of the hemodynamic variables showed statistically significant differences when comparing patients with none-to-mild FALD versus moderate-to-severe FALD (SVEDP: *p* = 0.34; mPAP: *p* = 0.87) (Table 2).

### 3.2. Perioperative Characteristics

The Fontan procedure was performed with CPB in 53.8% (n = 21) of patients, and aortic cross-clamping was used in 17.9% (n = 7). Myocardial protection was achieved using antegrade crystalloid cardioplegia with Custodiol^®^ solution in 5 cases (12.8%) and del Nido solution in 2 cases (5.1%), with no significant differences between groups (*p* = 0.99). The median CPB time length was 131 min (IQR 95–160), with no differences between patients with none-to-mild FALD and those with moderate-to-severe FALD (*p* = 0.10). Likewise, the median aortic cross-clamp time was 83 min (IQR 15–112), with no significant differences between groups (*p* = 0.85) (Table 3).

The procedure was performed using an extracardiac conduit in 97.4% of cases (n = 38), with a median conduit size of 18 mm (IQR 18–18.5), with no significant differences between groups (*p* = 0.19). Fenestration was performed in all cases, with an overall median size of 7 mm (IQR 5–8). Although patients with moderate-to-severe FALD had a smaller median fenestration size (5 mm [IQR 4.75–8]), this difference was not statistically significant (*p* = 0.12) (Table 3).

The median length of stay in PICU was 5 days (IQR 3–7.9), and the median time on mechanical ventilation was 34 h (IQR 15.5–98), with no significant differences according to FALD severity. The postoperative hemodynamic profile was comparable between patients with none-to-mild FALD versus moderate-to-severe FALD, with a median arterial oxygen saturation of 90% (IQR 85–94) (*p* = 0.55); a median systemic ventricular ejection fraction of 60% (IQR 56–60) (*p* = 0.41); median SVEDP, measured through the median of mean left atrial pressure of 10 mmHg (IQR 9–12) (*p* = 0.64); and a median of mean mPAP of 14 mmHg (IQR 12–17) (*p* = 0.55) (Table 3).

The overall median duration of chest tube drainage was 14.8 days (IQR 9.9–20.9), with no significant difference between moderate-to-severe and none-to-mild FALD (19.4 days [IQR 16.5–21.4] vs. 13.5 days [IQR 9.2–19.1]; *p* = 0.11). Chest drainage volume showed a nonsignificant inverse trend (252 mL/day/m^2^ [IQR 229–321.5] vs. 282 mL/day/m^2^ [IQR 233.5–373.5]; *p* = 0.41). The median duration of peritoneal drainage was 10.8 days (IQR 7.9–14.9), with no difference between groups (*p* = 0.55); drainage volume similarly showed a nonsignificant inverse trend (164 mL/day/m^2^ [IQR 87.5–348.5] vs. 265.5 mL/day/m^2^ [IQR 184–410]; *p* = 0.27) (Table 3).

### 3.3. Follow-Up and Assessment of Liver Disease

The median postoperative follow-up time was 9.2 years (IQR 6–12.1). At the time of MRI evaluation, the overall median age was 18.1 years (IQR 14.9–22.8). Patients with moderate-to-severe FALD were significantly older than those with none-to-mild FALD (23.3 years [IQR 15.3–28.5] vs. 17.8 years [IQR 14.3–21.7]; *p* = 0.14). Regarding clinical status, the majority of the cohort (97.4%, n = 38) were in NYHA functional class II (Table 3).

Cardiac MRI evaluation showed moderate atrioventricular valve regurgitation in 4 patients (10.2%) and severe regurgitation in 3 patients (7.7%), with no significant difference between groups (*p* = 0.56). Regarding systemic ventricular function and volumes, the cohort had a median ejection fraction of 52% (IQR 47.2–56), a median indexed end-systolic volume of 35.8 mL/m^2^ (IQR 30.1–41.9), and a median indexed end-diastolic volume of 74.4 mL/m^2^ (IQR 63–91.4), with no significant differences between the two groups (Table 3).

The evaluation of laboratory parameters revealed differences in several variables (Table 3). Platelet counts were lower in patients with moderate-to-severe FALD than in those with none-to-mild FALD, although this difference was not statistically significant (148 × 10^3^/µL [IQR 74.5–205.8] vs. 183 × 10^3^/µL [IQR 157–214]; *p* = 0.27). In contrast, AST levels were significantly higher in the moderate-to-severe FALD group (37.8 U/L [IQR 28.5–53.9] vs. 28.5 U/L [IQR 23.5–32.3]; *p* = 0.03). GGT levels were also higher in this group (61.1 U/L [IQR 44.2–150.5] vs. 44 U/L [IQR 31.2–70.5]), although the difference was not statistically significant (*p* = 0.27). Albumin concentrations were similar between the two groups (4.7 g/dL [IQR 4.1–5.1] vs. 4.7 g/dL [IQR 4.4–4.9]; *p* = 0.78). In contrast, PT was significantly longer in patients with moderate-to-severe FALD (18.4 s [IQR 12.2–28.1] vs. 12.7 s [IQR 12.3–13.9]; *p* < 0.001).

Abdominal ultrasonography findings demonstrated a greater burden of structural liver abnormalities in patients with moderate-to-severe FALD. Parenchymal fibrotic alterations differed significantly between groups (*p* = 0.01). Among patients with none-to-mild FALD, 8 (24.2%) had no fibrotic alterations, 24 (72.7%) had mild fibrosis, and 1 (3%) had moderate fibrosis. In contrast, all patients with moderate-to-severe FALD had fibrotic alterations: 4 (66.7%) had mild fibrosis and 2 (33.3%) had moderate fibrosis. No cases of severe fibrosis were identified in either group (Table 3).

A blunted liver edge was observed in 38 (97.4%) patients, with no difference between groups (97% vs. 100%; *p* = 0.99). Surface nodularity was more frequent in patients with moderate-to-severe FALD than in those with none-to-mild FALD (50% vs. 21.2%), although this difference was not statistically significant (*p* = 0.16). Splenomegaly was significantly more frequent in the moderate-to-severe FALD group (50% vs. 9.1%; *p* = 0.03). No patient had ascites (Table 3).

### 3.4. Assessment of Fontan-Associated Liver Disease Using Liver Magnetic Resonance Imaging Mapping

Analysis of quantitative liver MRI parameters showed an apparent between-group difference in T1 mapping at the level of segment I (Figure 3, Supplementary Table S1). Patients with moderate-to-severe FALD had longer T1 relaxation times than those with none-to-mild FALD (760 ± 71 ms vs. 708 ± 46 ms; unadjusted *p* = 0.03). No significant differences were observed in T1 relaxation times for segments II–VIII or in measurements of the left (*p* = 0.41) and right (*p* = 0.69) lobes. Likewise, T2 relaxation times and ADC showed similar patterns between groups across all evaluated segments and lobar averages, with no statistically significant differences. Overall, these findings should be considered exploratory, as the between-group difference in segment I T1 relaxation time did not remain statistically significant after adjustment for multiple comparisons.

Univariate logistic regression analysis did not identify any clinical, surgical, or hemodynamic predictors associated with greater liver disease severity (Table 4). Variables including age and weight at surgery, predominant ventricular morphology, Nakata index, McGoon ratio, use of CPB and aortic cross-clamp, fenestration size, systemic ventricular ejection fraction, atrioventricular valve regurgitation, and follow-up duration were evaluated, with no significant associations identified. Given the absence of significant univariate predictors and the sample size, multivariate analysis was not considered justified.

ROC curve analysis revealed heterogeneity in the discriminative performance of quantitative parameters across hepatic segments. T1 mapping in segment I showed the highest discriminative performance for differentiating between none-to-mild and moderate-to-severe FALD (AUC = 0.771; 95% CI: 0.500–1.000). The optimal cutoff, determined using the Youden index, was >734 ms (J = 0.583), yielding a sensitivity of 83.3% (95% CI: 43.65–99.15), specificity of 75% (95% CI: 57.89–86.75), and a positive likelihood ratio (LR+ = 3.33) (Figure 4, Supplementary Table S2). In contrast, the observed AUC values for both T2 mapping and ADC were below 0.66 in segment I. Across segments II–VIII, AUC values ranged from 0.500–0.656 for T2 mapping and from 0.456–0.648 for ADC, with no evidence of meaningful discriminative performance in this exploratory analysis.

## 4. Discussion

FALD is one of the most relevant extracardiac complications in patients with a univentricular heart who undergo palliation with the Fontan procedure [14,24,25]. Its pathogenesis originates from chronic central venous congestion and reduced cardiac output, which result in severe impairment of hepatic capillary microperfusion and prolonged tissue hypoxia [26]. Histologically, this hemodynamic stress induces sinusoidal dilation and perivenular fibrosis, progressively evolving toward a pattern that also involves remodeling of the portal space [16], ultimately leading to cardiac cirrhosis, portal hypertension, and the development of hyperplastic nodules with a risk of transformation into hepatocellular carcinoma [24].

Despite the progressive nature of tissue injury, the assessment of FALD is substantially limited by a marked clinicopathologic dissociation; routine biochemical tests frequently remain within normal limits, and hemodynamic parameters do not always reflect the extent of parenchymal remodeling [16,25]. In this regard, although advanced quantitative techniques, such as T1 mapping by MRI, have emerged as promising noninvasive tools for detecting diffuse parenchymal abnormalities and their correlation with clinical hepatic dysfunction [27], accurate staging of FALD and timely identification of advanced disease stages remain major challenges that require multimodal diagnostic strategies and standardized clinical consensus [14,24,25].

We agree with the findings reported by Hansen et al. [19] regarding the early onset of tissue injury preceding overt hepatic failure. Therefore, we evaluated clinical, biochemical, and imaging parameters in patients with Fontan circulation, seeking to establish their relationship with tissue findings on liver MRI and identify a quantitative cutoff for discriminating FALD severity, considering previous findings reported by other centers [14,17,19].

The overall median follow-up duration in our cohort was 9 years (Table 3), similar to that reported in previous series [14,19], and we found no significant differences in follow-up duration between patients with none-to-mild FALD and those with moderate-to-severe FALD (*p* = 0.41). However, the latter group had a slightly longer follow-up despite undergoing surgery at an older age. In addition, although patients with moderate-to-severe FALD showed a trend toward longer CPB times, larger extracardiac conduit size, and longer durations of chest tube drains and peritoneal drainage, none of the perioperative hemodynamic variables showed significant differences (Table 3), and the hemodynamic profiles, particularly mPAP and SVEDP, remained highly similar. These findings suggest that the development of FALD is not primarily determined by an acute surgical event or its immediate complications, but rather by the cumulative stress imposed by Fontan physiology over time. This observation is consistent with previous reports [16,25,28] showing a weak correlation between isolated surgical factors and the degree of hepatic fibrosis, supporting the concept that FALD is a multifactorial condition resulting from the interaction of chronic hemodynamic, inflammatory, lymphatic, and vascular remodeling mechanisms.

On the other hand, although fenestration size was smaller in the moderate-to-severe FALD group in our cohort, this difference was not statistically significant (*p* = 0.12). Although fenestration theoretically decompresses the systemic venous circulation at the expense of cyanosis secondary to relative hypoxemia, we did not observe a significant impact on oxygen saturation in our study (Table 3), nor was fenestration identified as an independent factor associated with the development of moderate-to-severe FALD (Table 4). This finding is consistent with that reported by Hansen et al. [19], suggesting that fenestration size is not a significant determinant of hepatic involvement at present. However, given that routine fenestration is universal in our center’s protocol, longitudinal follow-up and larger cohort expansion are warranted to definitively determine its long-term clinical relevance.

Segmental analysis of liver MRI mapping (T1, T2, and ADC) revealed a heterogeneous distribution of tissue abnormalities. The main finding was prolongation of T1 relaxation time, predominantly localized to segment I. This observation is consistent with recent reports [29] highlighting regional heterogeneity of the liver parenchyma in Fontan circulation among patients with a systemic ventricle and the need to standardize the anatomical regions used for measurement. From a pathophysiological perspective, the direct venous drainage of segment I into the inferior vena cava through the caudate veins, together with its dual vascular supply [21], makes this region hemodynamically vulnerable to systemic venous hypertension. This retrograde overload may promote hepatic congestion and expansion of the extracellular space, potentially contributing to prolonged T1 relaxation times. Thus, segment I may represent a potentially informative regional marker of tissue remodeling, although this hypothesis requires further investigation.

Importantly, a quantitative T1 cutoff of >734 ms in this segment demonstrated the highest discriminative performance among the evaluated MRI parameters, with a sensitivity of 83.3% (Figure 4). However, the between-group difference in T1 relaxation time in segment I did not remain statistically significant after adjustment for multiple comparisons. Furthermore, the proposed T1 threshold should be interpreted cautiously given the small number of patients with moderate-to-severe FALD included in the analysis and the wide confidence intervals associated with this estimate. Therefore, this threshold should be considered an exploratory, hypothesis-generating finding rather than a clinically validated cutoff. Further studies in larger and independent cohorts, including internal and external validation, are required to assess its reproducibility, diagnostic performance, and potential clinical utility.

In contrast, no significant differences were observed in T2 relaxation times or ADC. These findings suggest that, in the early stages of FALD, microstructural abnormalities detectable by T1 mapping precede changes associated with edema, inflammation, or diffusion restriction [17,29]. It is possible that extracellular space expansion and early collagen deposition alter T1 relaxation times earlier than parameters related to tissue water content (T2) or molecular water mobility (ADC), which may explain the greater sensitivity of T1 mapping in subclinical stages. Although the sample size may have limited the statistical power to detect differences in T2 and ADC, the differential behavior of T1, T2, and ADC supports the potential utility of T1 mapping.

Taken together, these findings support the hypothesis that the first years following Fontan procedure represent a critical window of opportunity for establishing structured surveillance and early identification of progressive structural changes in the hepatic parenchyma. Nevertheless, defining the optimal time to initiate such surveillance will require prospective, multicenter studies before its formal incorporation into clinical practice guidelines.

We recognize the limitations of our study. First, the retrospective, single-center, nonrandomized design may limit the generalizability of our findings to other patient populations and clinical settings. Second, the sample size was limited due to the recent implementation of this liver MRI assessment protocol at our institution, in addition to geographic dispersion and socioeconomic barriers that hinder continuous follow-up at our tertiary care center, resulting in some patients receiving care at their primary healthcare centers. The limited sample size, particularly the small number of patients with advanced FALD, limits the statistical power of the study and warrants cautious interpretation of the results. Therefore, these findings should be considered exploratory and hypothesis-generating. Third, although a comprehensive set of clinical, laboratory, and imaging variables was included, other variables that may have influenced the outcome may not have been considered. Additionally, contemporaneous invasive hemodynamic data were not available, as routine cardiac catheterization is not performed in clinically stable Fontan patients at our institution for surveillance or research purposes. This precludes direct correlation of MRI-derived FALD measures with current invasive hemodynamic parameters, although the non-invasive nature of the MRI-based assessment was consistent with the primary objective of this study. Finally, long-term follow-up is required to determine the definitive impact of duration of exposure on disease progression. Despite these limitations, this study represents a valuable initial approach and provides relevant scientific evidence regarding the behavior of FALD in our region.

## 5. Conclusions

After a decade of follow-up, approximately 15% of patients had moderate-to-severe FALD. In addition to biochemical parameters and abdominal ultrasonography, liver MRI may provide complementary information for the noninvasive assessment of FALD. Notably, prolonged T1 relaxation time in segment I (>734 ms) showed the highest discriminative performance among the evaluated MRI parameters for identifying moderate-to-severe FALD, with a sensitivity exceeding 80%. However, this threshold should be considered exploratory and requires further validation in larger, independent cohorts. The temporal variability of T2 and ADC, together with the multifactorial nature of FALD, underscores the need for long-term, multimodal, noninvasive follow-up.

Improving screening strategies during follow-up of patients with Fontan circulation is essential for the early detection of progressive liver changes and for implementing measures aimed at improving quality of life and overall survival.

## Figures and Tables

**Figure 1:**
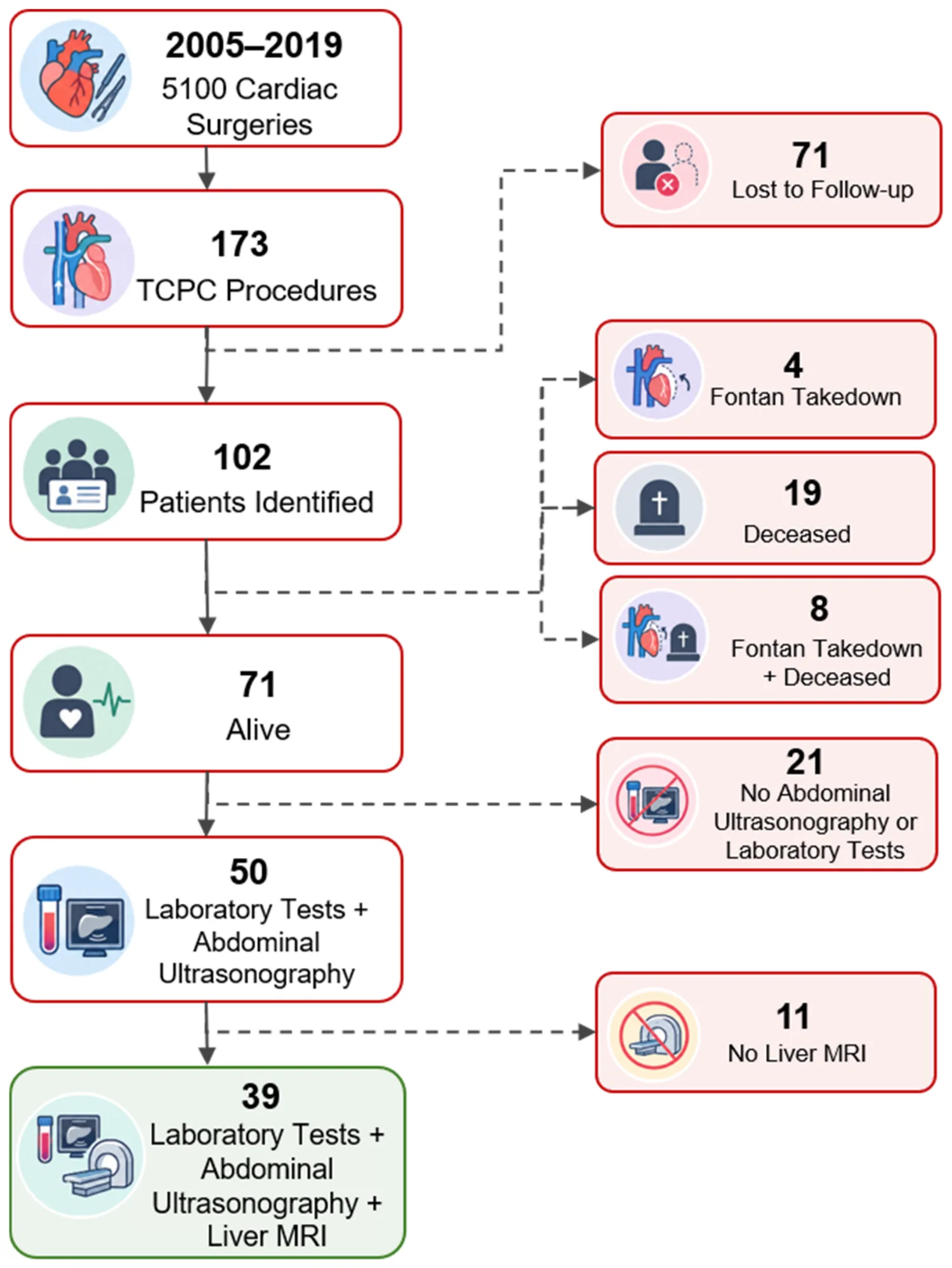
Flowchart of patient selection, follow-up, and exclusion criteria for the final study cohort. TCPC: total cavopulmonary connection, MRI: magnetic resonance imaging.

**Figure 2:**
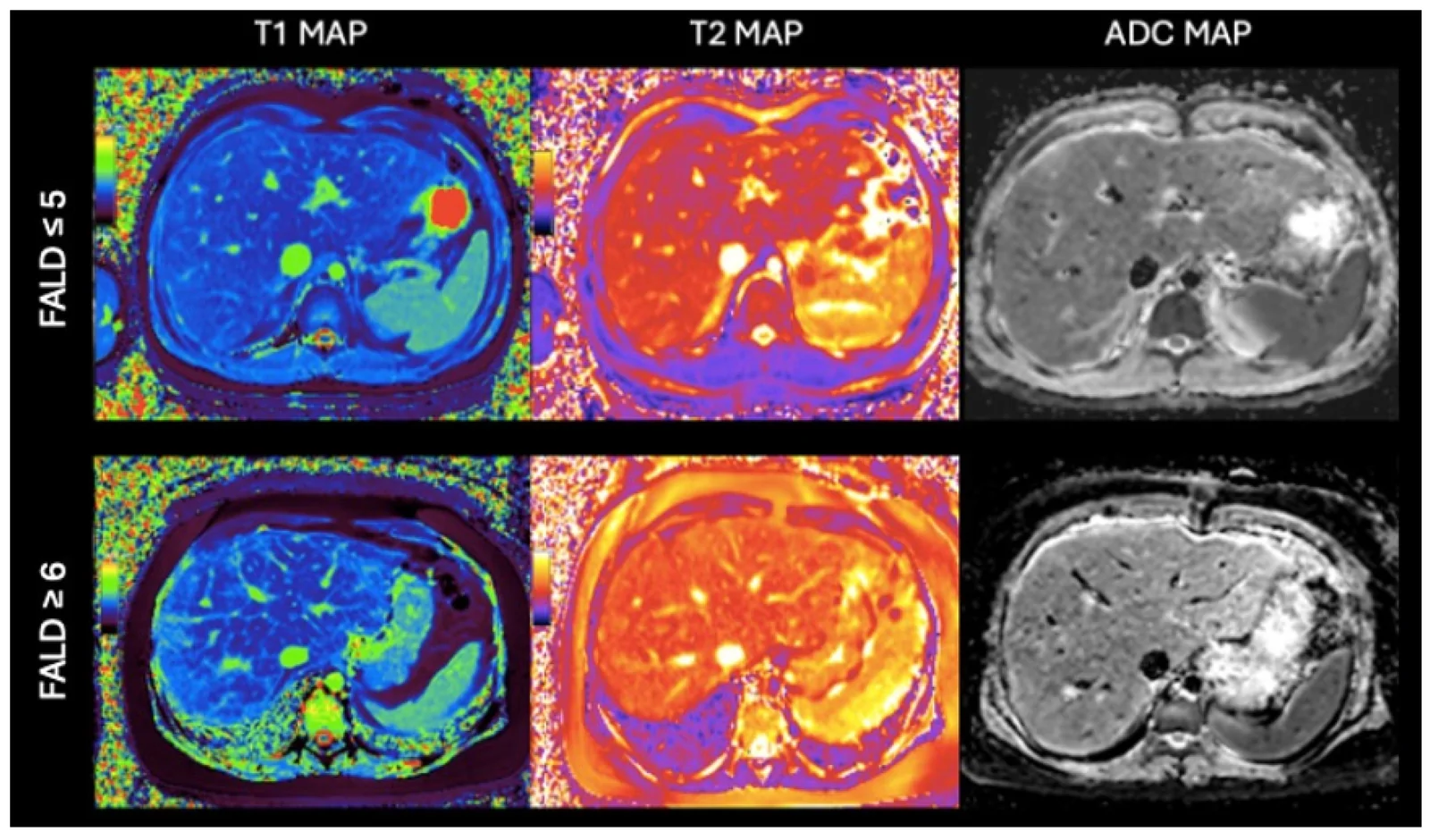
Representative liver MRI mapping, including ADC, T1, and T2 mapping, in a patient with no FALD (upper row) and a patient with moderate FALD (lower row). Compared with the patient with no FALD, the patient with moderate FALD shows prolonged T1 and T2 relaxation times, with corresponding changes in the color appearance of the parametric maps. The ADC map also demonstrates greater spatial heterogeneity of diffusion values throughout the hepatic parenchyma. ADC: apparent diffusion coefficient, FALD: Fontan-associated liver disease.

**Figure 3:**
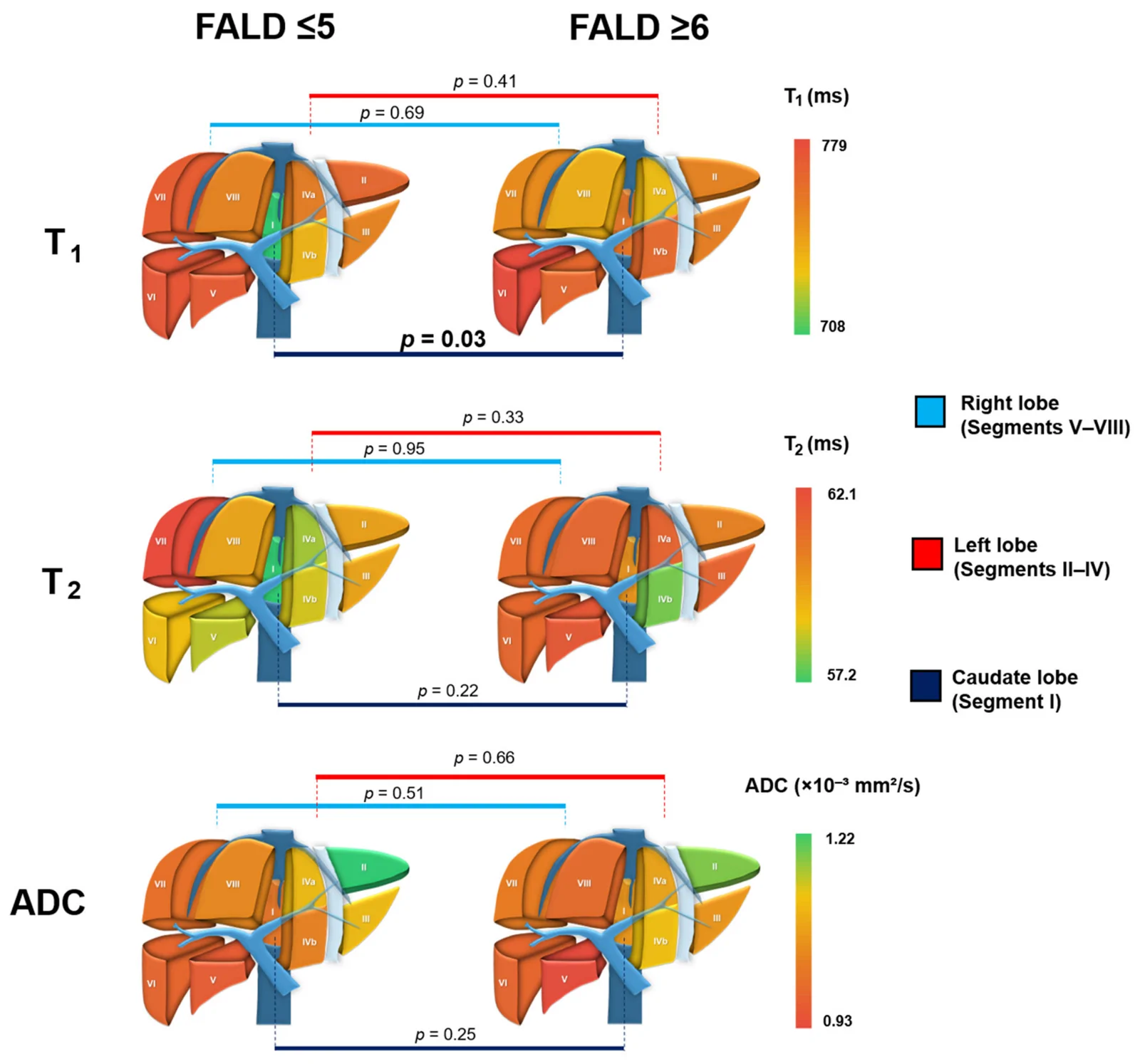
Segmental analysis of T1, T2, and ADC mapping. Color-coded segmental T1, T2, and ADC values. Significant differences were observed in T1 mapping at the level of segment I. ADC: apparent diffusion coefficient, FALD: Fontan-associated liver disease.

**Figure 4:**
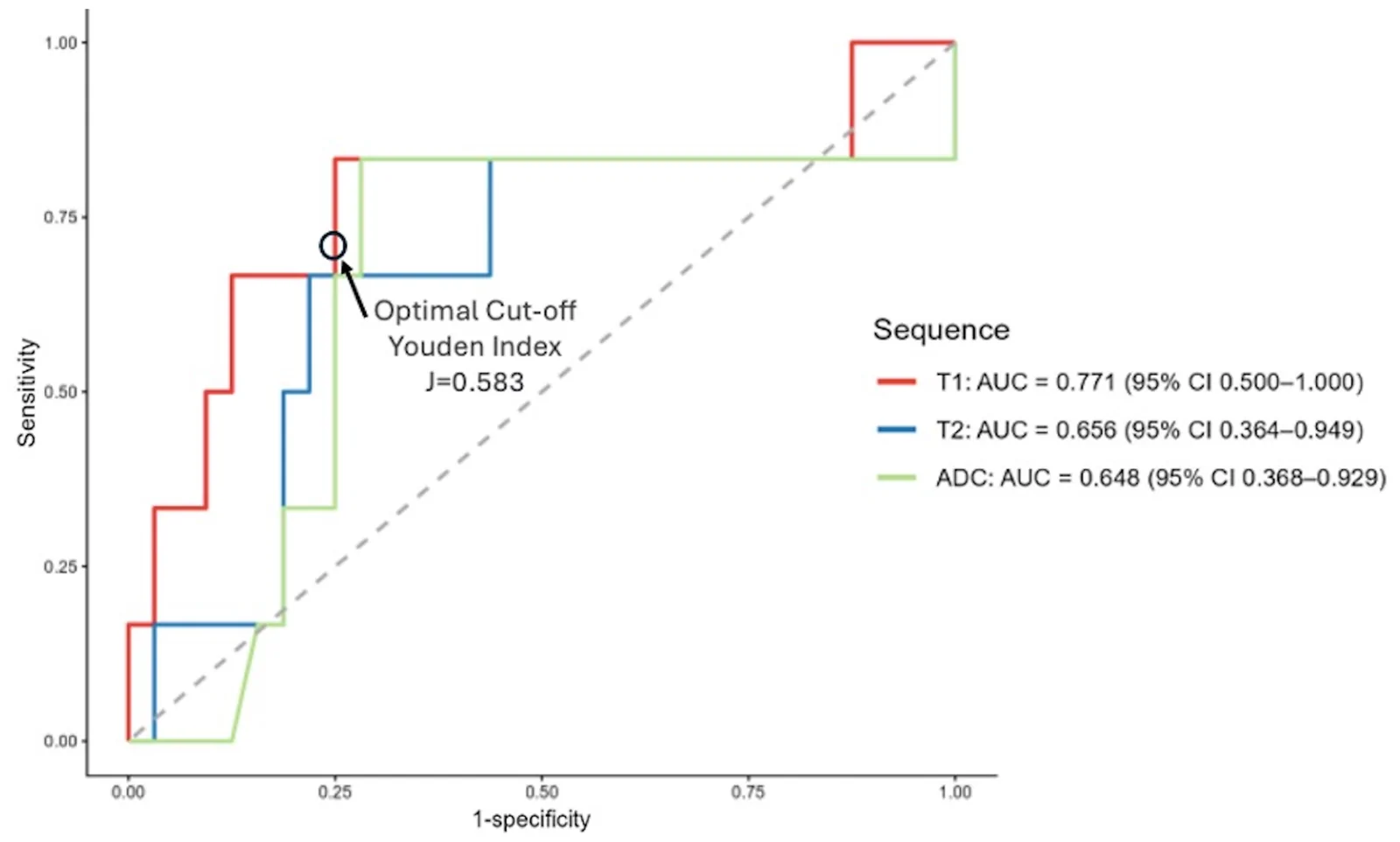
Discriminative performance of MRI parameters in segment I for differentiating between none-to-mild and moderate-to-severe FALD. ROC curves for T1 (red; AUC = 0.771), T2 (blue; AUC = 0.656), and ADC (green; AUC = 0.648). For T1, the optimal cutoff according to the Youden index was >734 ms, with a sensitivity of 83.3% and specificity of 75% (J = 0.583, LR+ = 3.33). Dashed line: reference AUC of 0.5. ADC: apparent diffusion coefficient, FALD: Fontan-associated liver disease.

**Table 1:** Distribution of patients according to FALD severity, diagnoses and pre-Fontan palliative procedures.

Characteristics, n (%)	Total 39 (100)
**FALD severity, n (%)**	
None (0–2)	8 (20.5)
Mild (3–5)	25 (64.1)
Moderate (6–8)	6 (15.4)
**Diagnosis, n (%)**	
Tricuspid atresia	20 (51.3)
DORV	5 (12.8)
Pulmonary atresia with intact interventricular septum	2 (5.1)
TGA	2 (5.1)
Unbalanced AV channel	1 (2.6)
Others	9 (23.1)
**Fontan palliative procedures n (%)**	
Systemic-to-pulmonary shunt	19 (48.7)
Bidirectional cavopulmonary connection	23 (58.9)
Pulmonary artery banding	4 (10.2)

AV: atrioventricular, DORV: double-outlet right ventricle, FALD: Fontan-associated liver disease, TGA: transposition of the great arteries.

**Table 2:** Overall characteristics of patients according to FALD severity.

Characteristics, n (%)	Total 39 (100)	FALD ≤ 5 33 (84.6)	FALD ≥ 6 6 (15.4)	*p*
**Sex, n (%)**				
Male	15 (38.5)	13 (39.4)	2 (33.3)	0.99
Female	24 (61.5)	20 (60.6)	4 (66.7)	
**Age at surgery (years), median (IQR)**	9.3 (5.9–10.8)	8.9 (5.8–10.7)	10.6 (9.6–12)	0.06
**Weight at surgery (kg), median (IQR)**	25 (18.5–37.5)	24 (17.1–36)	39 (24.7–43.2)	0.05
**BSA at surgery (m^2^), median (IQR)**	0.9 (0.8–1.2)	0.9 (0.7–1.2)	1.2 (0.9–1.3)	0.18
**Preoperative NYHA class, n (%)**				
I	1 (2.6)	1 (3)	0 (0)	0.51
II	30 (76.9)	26 (78.8)	4 (66.7)	
III	7 (17.9)	5 (15.2)	2 (33.3)	
IV	1 (2.6)	1 (3)	0 (0)	
**Previous surgeries, n (%)**				
0	5 (12.8)	4 (12.1)	1 (16.7)	0.99
1	22 (56.4)	19 (57.6)	3 (50)	
2	12 (30.8)	10 (30.3)	2 (33.3)	
**Pre-Fontan surgical procedures, n (%)**				
Systemic-to-pulmonary shunt	19 (48.7)	15 (45.5)	4 (66.7)	0.41
Bidirectional cavopulmonary connection	23 (58.9)	20 (60.6)	3 (50)	0.67
**Predominant ventricular morphology, n (%)**				
Left ventricle	29 (74.4)	25 (75.7)	4 (66.7)	0.13
Right ventricle	6 (15.4)	6 (18.2)	0 (0)	
Undetermined	4 (10.3)	2 (6.1)	2 (33.3)	
**Preoperative anatomical/hemodynamic conditions**				
Arterial oxygen saturation (%), median (IQR)	90 (85–94)	90 (85–94.5)	86.5 (85–92.5)	0.53
Systemic ventricular ejection fraction (%), median (IQR) *	61 (60–64)	61 (60–64.1)	60 (55.9–62)	0.16
Systemic ventricular end-diastolic pressure (mmHg), median (IQR)	8 (7–10)	8 (7–9.5)	9 (7.7–10.5)	0.34
Mean pulmonary artery pressure (mmHg), median (IQR)	12 (10–13)	12 (10–13.5)	12 (10–13.5)	0.87
Nakata index (mm^2^/m^2^), median (IQR)	230 (200–297)	239 (196–298)	229 (196–340)	0.89
McGoon ratio, median (IQR)	2.1 (1.8–2.2)	2.1 (1.8–2.2)	2.0 (1.6–2.2)	0.74

BSA: body surface area, IQR: interquartile range, NYHA: New York Heart Association, SD: standard deviation. * Assessed by transthoracic echocardiogram.

**Table 3:** Operative, postoperative, and follow-up characteristics according to FALD severity.

Characteristics, n (%)	Total 39 (100)	FALD ≤ 5 33 (84.6)	FALD ≥ 6 6 (15.4)	*p*
**Surgical characteristics**				
**Surgical approach, n (%)**				
With CPB	21 (53.8)	17 (51.5)	4 (66.7)	0.68
Without CPB	18 (46.2)	16 (48.5)	2 (33.3)	
**CPB and aortic cross-clamping**				
Time of CPB (min), median (IQR)	131 (95–160)	106 (90.5–148)	167.5 (132.3–233.5)	0.10
Time of aortic cross-clamp (min), median (IQR)	83 (15–112)	66 (12.5–143.5)	90.5 (83–98)	0.85
**Cardioplegia type, n (%)**				0.99
Custodiol	5 (12.8)	4 (12.1)	1 (16.7)	
del Nido	2 (5.1)	1 (3)	1 (16.7)	
**Fontan type, n (%)**				
Extracardiac TCPC	38 (97.4)	33 (100)	5 (83.3)	0.15
Intracardiac TCPC	1 (2.6)	0 (0)	1 (16.7)	
**Conduit size (mm), median (IQR)**	18 (18–18.5)	18 (18–18)	20 (17–20)	0.19
**Fenestra size (mm), median (IQR)**	7 (5–8)	7 (6–8)	5 (4.75–8)	0.12
**Post-operative characteristics**				
**PICU length of stay (days), median (IQR)**	5 (3–7.9)	5 (2.9–7.8)	5.3 (3.5–10.9)	0.68
**Mechanical ventilation time (h), median (IQR)**	34 (15.5–98)	30 (12.8–97.5)	60 (26.7–133.9)	0.43
**Maximum lactate (mmol/L), median (IQR)**	3.2 (2.4–4.5)	3.1 (2.3–4.5)	3.4 (2.9–4.5)	0.47
**Post-operative hemodynamic conditions**				
Arterial oxygen saturation (%), median (IQR)	90 (85–94)	90 (85–94.5)	86.5 (85–92.5)	0.55
Systemic ventricular ejection fraction (%), median (IQR) *	60 (56–60)	60 (55–60)	60 (57.5–60.5)	0.41
Mean left atrial pressure (mmHg), median (IQR)	10 (9–12)	10 (8.5–12)	10 (9–10.2)	0.64
Mean pulmonary artery pressure (mmHg), median (IQR)	14 (12–17)	14 (12–17)	15 (13–15)	0.55
**Chest tube drains**				
Drainage time (days), median (IQR)	14.8 (9.9–20.9)	13.5 (9.2–19.1)	19.4 (16.5–21.4)	0.11
Volume (mL/day/m^2^), median (IQR)	273 (234–365)	282 (233.5–373.5)	252 (229–321.5)	0.41
**Peritoneal drainage**				
Drainage time (days), median (IQR)	10.8 (7.9–14.9)	10.7 (7.9–14.9)	11.9 (8.9–18.4)	0.55
Volume (mL/day/m^2^), median (IQR)	258 (175–409)	265.5 (184–410)	164 (87.5–348.5)	0.27
**Follow-up characteristics**				
**Age at MRI (years), median (IQR)**	18.1 (14.9–22.8)	17.8 (14.3–21.7)	23.3 (15.3–28.5)	0.14
**Weight at MRI (kg), median (IQR)**	55 (48–65)	54 (47.5–62.2)	64.5 (54.7–73)	0.05
**BSA at MRI (m^2^), median (IQR)**	1.6 (1.5–1.7)	1.6 (1.5–1.7)	1.6 (1.6–1.8)	0.11
**NYHA class at follow-up, n (%)**				
I	1 (2.6)	1 (3)	0 (0)	0.99
II	38 (97.4)	32 (97)	6 (100)	
**Functional conditions assessed through MRI**				
**Atrioventricular valve regurgitation, n (%)**				
None	17 (43.6)	15 (45.5)	2 (33.3)	0.56
Mild	15 (38.5)	11 (33.3)	4 (66.7)	
Moderate	4 (10.2)	4 (12.1)	0 (0)	
Severe	3 (7.7)	3 (9.1)	0 (0)	
Systemic ventricular ejection fraction (%), median (IQR) **	52 (47.2–56)	52 (47.7–56.2)	49 (44.2–52.7)	0.34
**ESV indexed** **—** **systemic ventricle (mL/m^2^), median (IQR)**	35.8 (30.1–41.9)	35.8 (30.4–41.1)	36.2 (21.6–44.7)	0.95
**EDV indexed** **—** **systemic ventricle (mL/m^2^), median (IQR)**	74.4 (63–91.4)	76.9 (65.8–93.2)	66.4 (51.2–76)	0.15
**Liver disease score**				
**Laboratory data**				
Platelet count (×10^3^/µL), median (IQR)	183 (148.8–214)	183 (157–214)	148 (74.5–205.8)	0.27
AST (U/L), median (IQR)	29.3 (24.9–34.7)	28.5 (23.5–32.3)	37.8 (28.5–53.9)	0.03
GGT (U/L), median (IQR)	44.9 (32–74)	44 (31.2–70.5)	61.1 (44.2–150.5)	0.27
Albumin (g/dL), median (IQR)	4.7 (4.4–4.9)	4.7 (4.4–4.9)	4.7 (4.1–5.1)	0.78
Prothrombin time (s), median (IQR)	12.9 (12.3–14.2)	12.7 (12.3–13.9)	18.4 (12.2–28.1)	<0.001
**Abdominal ultrasonography findings, n (%)**				
**Parenchymal fibrotic changes of the liver**				
Absent	8 (20.5)	8 (24.2)	0 (0)	0.01
Mild	28 (71.8)	24 (72.7)	4 (66.7)	
Moderate	3 (7.7)	1 (3)	2 (33.3)	
Severe	0 (0)	0 (0)	0 (0)	
**Blunted liver edge**	38 (97.4)	32 (97)	6 (100)	0.99
**Surface nodularity**	10 (25.6)	7 (21.2)	3 (50)	0.16
**Splenomegaly**	6 (15.4)	3 (9.1)	3 (50)	0.03
**Ascites**	0 (0)	0 (0)	0 (0)	
**Follow-up after Fontan (years), median (IQR)**	9.2 (6–12.1)	8.3 (6–11.8)	12.4 (5.7 –17.3)	0.41

BSA: body surface area, CPB: cardiopulmonary bypass, EDV: end-diastolic volume, ESV: end-systolic volume, IQR: interquartile range, MRI: magnetic resonance imaging, NYHA: New York Heart Association, PICU: pediatric intensive care unit, SD: standard deviation, TCPC: total cavopulmonary connection. * Assessed by transthoracic echocardiogram, ** Assessed by magnetic resonance imaging.

**Table 4:** Univariate analysis of factors associated with moderate-to-severe FALD.

Variable	OR	95% CI		*p*
		Lower	Upper	
Age at surgery (years)	0.19	0.03	1.09	0.05
Weight at surgery (kg)	0.43	0.09	1.79	0.05
Predominant ventricular morphology	1.55	0.37	6.75	0.68
Nakata index (mm^2^/m^2^)	1.9	0.46	8.26	0.95
McGoon ratio	1.39	0.34	6.08	0.75
Use of CPB	1.71	0.41	7.47	0.67
Use of aortic cross-clamping	2	0.39	9.53	0.57
Fenestration size (mm)	1.12	0.28	4.89	0.12
Systemic ventricular ejection fraction at follow-up (%) **	3.57	0.64	22.13	0.12
Atrioventricular valve regurgitation at follow-up **	0.56	0.13	2.29	0.66
Follow-up after surgery (years)	0.41	0.07	2.09	0.41

CI: confidence interval, CPB: cardiopulmonary bypass, OR: odds ratio. ** Assessed by magnetic resonance imaging.

## Data Availability

The raw data supporting the conclusions of this article will be made available by the authors on request.

## Supplementary Materials

The following supporting information can be downloaded at: https://www.mdpi.com/article/10.3390/diagnostics16213512/s1: Supplementary Table S1: Liver T1 and T2 relaxation times and apparent diffusion coefficient according to FALD severity; Supplementary Table S2: Diagnostic performance of segment I T1 relaxation time for identifying moderate-to-severe FALD.

## Abbreviations

The following abbreviations are used in this manuscript:

ADC	Apparent diffusion coefficient
AST	Aspartate aminotransferase
AUC	Area under the curve
CPB	Cardiopulmonary bypass
ECG	Electrocardiographic
FALD	Fontan-associated liver disease
GGT	Gamma-glutamyl transferase
IQR	Interquartile range
LDS	Liver disease score
MRI	Magnetic resonance imaging
OR	Odds ratio
PT	Prothrombin time
ROC	Receiver operating characteristic
T1	T1 relaxation time
T2	T2 relaxation time
