# Supplementary material for: Comprehensive Evaluation of Fontan-Associated Liver Disease: The Role of Magnetic Resonance Imaging
Source: Diagnostics (Basel). 2026 Oct 27;16(21):3512. doi: 10.3390/diagnostics16213512 (PMC13761829; doi:10.3390/diagnostics16213512)
Supplement: Supplementary file 1 [file diagnostics-16-03512-s001.zip › diagnostics-4550962-supplementary.pdf]

Supplementary Table S1. Liver T1 and T2 relaxation times and ADC according to FALD severity.

Parameter	Total 39 (100)	FALD ≤ 5 33 (84.6)	FALD ≥ 6 6 (15.4)	*p*
**T1 relaxation time (ms), mean ± SD**				
Segment I	716 ± 53	708 ± 46	760 ± 71	**0.03**
Segment II	764 ± 71	766 ± 67	754 ± 96	0.95
Segment III	754 ± 53	754 ± 53	755 ± 57	0.9
Segment IVa	752 ± 65	756 ± 66	734 ± 63	0.69
Segment IVb	741 ± 55	737 ± 47	765 ± 88	0.21
Segment V	771 ± 63	772 ± 65	766 ± 61	0.69
Segment VI	775 ± 53	774 ± 53	779 ± 55	0.77
Segment VII	768 ± 62	771 ± 63	751 ± 59	0.45
Segment VIII	752 ± 73	754 ± 75	739 ± 66	0.67
Left lobe	762 ± 49	762 ± 46	764 ± 68	0.41
Right lobe	787 ± 64	792 ± 61	763 ± 77	0.69
**T2 relaxation time (ms), mean ± SD**				
Segment I	57.6 ± 5.4	57.2 ± 5.1	60 ± 7	0.22
Segment II	59.8 ± 6.1	59.7 ± 5.8	60.4 ± 8	0.98
Segment III	59.9 ± 5.5	59.6 ± 4.9	61.3 ± 8.8	0.43
Segment IVa	58.7 ± 5.8	58.2 ± 5.4	61.3 ± 7.5	0.29
Segment IVb	58.5 ± 5.1	58.6 ± 4.9	57.8 ± 6.5	0.42
Segment V	58.9 ± 5.4	58.4 ± 5.2	61.7 ± 6	0.24
Segment VI	59.3 ± 6	59 ± 5.9	61.2 ± 6.9	0.59
Segment VII	61.9 ± 7.8	62.1 ± 7.6	61.1 ± 10	0.84
Segment VIII	59.8 ± 6.5	59.6 ± 6	61.2 ± 10.3	0.54
Left lobe	60.2 ± 5.4	59.9 ± 5	61.9 ± 7.5	0.33
Right lobe	60.4 ± 5.4	60.3 ± 5	61.2 ± 8	0.95
**ADC ($x10^{-3}$ mm^2/s), mean ± SD**				
Segment I	1 ± 0.2	1 ± 0.2	1.1 ± 0.2	0.25
Segment II	1.2 ± 0.2	1.2 ± 0.2	1.2 ± 0.1	0.78
Segment III	1.1 ± 0.2	1.1 ± 0.2	1.1 ± 0.2	0.67
Segment IVa	1.1 ± 0.2	1.1 ± 0.2	1.1 ± 0.3	0.52
Segment IVb	1.1 ± 0.2	1 ± 0.2	1.1 ± 0.3	0.29
Segment V	0.9 ± 0.2	0.9 ± 0.2	0.9 ± 0.2	0.84
Segment VI	0.9 ± 0.1	0.9 ± 0.1	0.9 ± 0.1	0.99
Segment VII	1 ± 0.2	1 ± 0.2	1 ± 0.2	0.96
Segment VIII	1 ± 0.2	1 ± 0.2	0.9 ± 0.1	0.77
Left lobe	1.1± 0.2	1.1 ± 0.2	1.1 ± 0.2	0.66
Right lobe	1 ± 0.2	1 ± 0.2	1 ± 0.1	0.51

ADC: apparent diffusion coefficient, **SD:** standard deviation.

Supplementary Table S2. Diagnostic performance of segment I T1 relaxation time for identifying moderate-to-severe FALD.

Parameter	Value
**Optimal cutoff**	>734 ms
**Youden index (J)**	0.583
**Sensitivity**	83.3% (95% CI 43.65–99.15)
**Specificity**	75 % (95% CI 57.89–86.75)
**Positive likelihood ratio (LR+)**	3.33
